# Emerging Role of microRNAs and Long Non-Coding RNAs in Sjögren’s Syndrome

**DOI:** 10.3390/genes12060903

**Published:** 2021-06-11

**Authors:** Giada De Benedittis, Cinzia Ciccacci, Andrea Latini, Lucia Novelli, Giuseppe Novelli, Paola Borgiani

**Affiliations:** 1 Department of Biomedicine & Prevention, Genetics Section, University of Rome Tor Vergata, 00133 Rome, Italy; dbngdi01@uniroma2.it (G.D.B.); a.latini@med.uniroma2.it (A.L.); novelli@med.uniroma2.it (G.N.); borgiani@med.uniroma2.it (P.B.); 2 UniCamillus–Saint Camillus International University of Health Sciences, 00131 Rome, Italy; lnovelli@hotmail.it; 3 IRCCS Neuromed, 86077 Pozzilli, Italy; 4 Department of Pharmacology, School of Medicine, University of Nevada, Reno, NV 89557, USA

**Keywords:** Sjögren’s Syndrome, microRNAs, long non-coding RNAs

## Abstract

Sjögren’s Syndrome (SS) is a chronic autoimmune inflammatory disease. It is considered a multifactorial pathology, in which underlying genetic predisposition, epigenetic mechanisms and environmental factors contribute to development. The epigenetic regulations represent a link between genetic predisposition and environmental factors. Recent studies suggested a regulatory role for non-coding RNAs in critical biological and disease processes. Among non-coding RNAs, microRNAs (miRNAs) and long non-coding RNAs (lncRNAs) play a critical role in the post-transcriptional mRNA expression, forming a complex network of gene expression regulation. This review aims to give an overview of the latest studies that have investigated the role of miRNAs and lncRNAs in the SS. We included papers that investigated the expression of non-coding RNAs on different tissues, in particular on peripheral blood mononuclear cells and salivary glands. However, regarding the involvement of non-coding RNAs genetic variability in SS susceptibility very few data are available. Further research could help to elucidate underlying pathogenic processes of SS and provide new opportunities for the development of targeted therapies.

## 1. Introduction

Sjögren’s syndrome (SS) is a chronic autoimmune inflammatory disease that typically affects the exocrine glands of the body, in particular lacrimal and salivary glands, causing dryness. This syndrome affects mainly females and the peak age of SS diagnosis is around 50 years. The progressive loss of glandular secretion is caused by the infiltration of lymphocytes into the exocrine glands, with patients complaining of dry eyes and dry mouth over time [1]. This chronic lymphocytic infiltration can be evaluated with a minor salivary gland biopsy, which can help in the diagnosis [2]. The most common symptoms are thus keratoconjunctivitis sicca, xerostomia and parotid gland swelling, but any organ can be involved with subsequent systemic manifestations (e.g., arthralgias/arthritis or kidney and lung involvement) [3]. Traditionally, SS can be distinguished into primary and secondary disease: the primary disease can be diagnosed in a patient with sicca syndrome in the absence of other underlying rheumatic diseases, and is often associated with anti-Ro/SS-A and anti-La/SSB autoantibodies. The secondary form is linked to other accompanying rheumatic diseases (frequently rheumatoid arthritis) [4]. Primary SS diagnosis can be tricky; thus, classification criteria were developed by the American College of Rheumatology (ACR) and the European League Against Rheumatism (EULAR) [5]. These criteria apply to any patient with at least one symptom of ocular or oral dryness and who has a score ≥4 when summing the counts from: labial salivary gland with focal lymphocytic sialadenitis and focus score ≥1.3, presence of anti-SSA (Ro), ocular staining score ≥5 (or van Bijsterfeld score ≥4) on at least one eye, Schirmer test ≤5 mm/5 min on at least one eye, unstimulated whole saliva flow rate ≤0.1 mL/min [5]. 

SS is a multifactorial disorder, with a mostly unknown etiology, which arises from a complex interplay of genetic, epigenetic, and environmental factors [6].

The latter include also viral infection (Epstein-Barr virus, cytomegalovirus, HCV, herpes virus) because the persistence of viral genetic material seems to be able to alter epithelial cell biologic properties of the glands, with consequent overexpression of type I IFN-inducible genes, resulting in inflammatory infiltration and tissue damage. 

The presence of a predisposed genetic background has been documented in several studies. Genes involved in both innate and adaptive immunity play a crucial role into the susceptibility toward the disease with HLA locus disclosing the strongest association [7,8]. 

**Figure 1:**
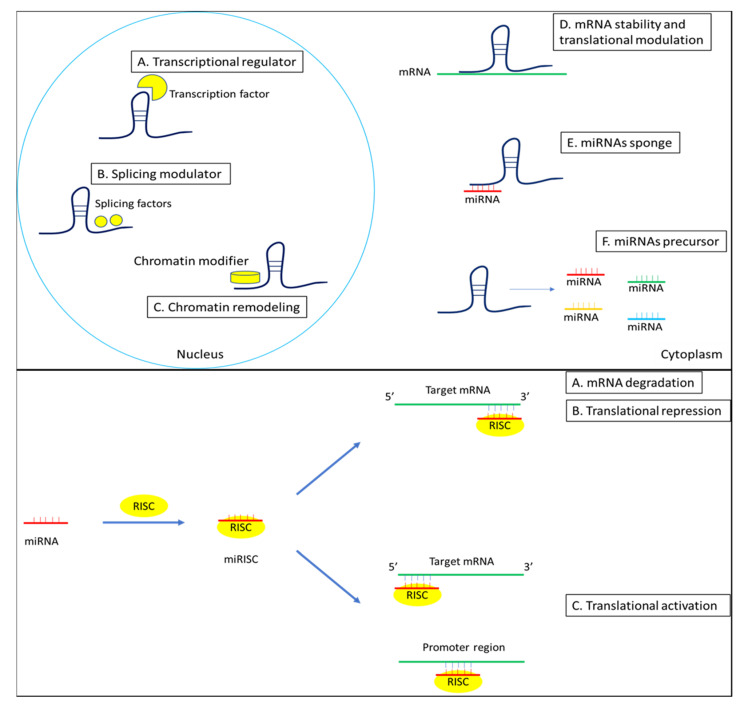
LncRNA (**above**) and miRNA (**below**) mechanisms of action in regulation of gene expression.

Epigenetic modifications have also emerged as important mechanisms for understanding how the interaction of genetic predisposition and environmental factors may give rise to chronic autoimmune diseases, including SS. Among the mechanisms of epigenetic regulation, we have non-coding RNAs (including microRNA and long non-coding RNA), a cluster of RNAs that do not encode functional proteins, but are considered regulatory RNAs; in fact, they play critical roles in the post-transcriptional mRNA expression (Figure 1). 

MicroRNAs (miRNAs) play a key role in gene silencing because, through the mechanism of RNA interference, they act as negative modulators of gene expression. They exert this function by binding to complementary sequences of their target mRNA in the 3’UTR region. In this way, they induce translational repression or destabilization of the target mRNA (Figure 1). In addition, miRNAs could also bind the 5’UTR region and the promoter region, inducing translational activation [9]. A key role of microRNAs in the regulation of the immune response has been highlighted, so it is not surprising that aberrant expression of miRNAs has been demonstrated in numerous autoimmune diseases [10,11], included SS. 

Another class of regulatory non-coding RNA are long non-coding RNAs (lncRNAs), recently recognized to play a role in the development of some autoimmune diseases [12]. LncRNAs are generally longer than 200 nucleotides and are located in the nucleus or cytoplasm. It is well defined that they play a significant role in epigenetic regulation, transcription, and translation; studies have shown that lncRNAs are involved in biological processes such as proliferation, differentiation, apoptosis, and immune responses. In particular, they are involved in the regulation of immune cell differentiation, activation, and responses. The mechanism of action of lncRNAs is still unclear, but it was observed that lncRNAs can act with other non-coding RNAs, including miRNAs (Figure 1). In fact, lncRNAs can act as “miRNA-sponge”: they can bind miRNAs, preventing their regulation on the mRNAs. In addition, lncRNAs can bind some miRNA’s targets because they compete with the 3’UTR and this bond can inhibit indirectly the negative regulation of mRNAs [13]. 

LncRNAs and miRNAs can interact with each other, regulating each other, forming a complex network of gene expression regulation. Through this multi-level regulation, these two non-coding RNA families are involved in several processes, included immune response. Several studies showed the role of these non-coding RNAs in the pathogenesis of several inflammatory diseases, including also SS. In particular, recent studies have highlighted the emerging role of lncRNAs, linking them with the regulation of inflammatory responses, the function of pro-inflammatory cytokines and the MHC protein complex. 

In light of this background, we decided to carry out this review, in order to provide an overview of the latest studies analyzing the role of miRNAs and lncRNAs in the SS.

## 2. Literature Search Strategy

The scientific publications were identified by the international web database PubMed. We selected the papers that investigated the role of miRNAs and lncRNAs in SS pathogenesis, focusing on the expression and genetic variability of these molecules. We included the most recent articles published up to 2020. We performed the research using the following key words: Sjogren’s syndrome, expression, long non-coding RNA or lncRNA and microRNA or miRNA, polymorphism or SNPs or genetic variant. Furthermore, we repeated the research focusing our attention on the identified miRNAs and lncRNAs. References of all selected articles were investigated. We included all observational population-based studies, which were conducted as case-control, cohort, and cross sectional analysis. On the other hand, we excluded animal studies, clinical trials, short communications, letters to the editor, dissertations, and in vitro studies. According to these criteria, we retrieved about 25 papers, which were all taken into consideration for this review. 

## 3. Roles of miRNAs in SS

Among non-coding RNAs, miRNAs are the most investigated. Due to their involvement in various crucial regulatory functions, any change in expression level could have an impact on target molecules. A single miRNA targets multiple downstream molecules and a single target gene may be regulated by multiple miRNAs. These studies were conducted on different types of samples, such as peripheral blood mononuclear cells (PBMCs), salivary glands, saliva, etc. Many dysregulated miRNAs have been found in SS patients.

### 3.1. miRNAs Dysregulated in PBMCs

The most investigated miRNA that is known to have a role in innate and adaptive immunity by negatively regulating the inflammatory response is hsa-miR-146 [14]. Pauley KM et al. studied the hsa-miR-146a expression in PBMCs of SS patients [15]. They showed that this miRNA is up-regulated in SS patients. They also elucidated the role of hsa-miR-146a in SS pathogenesis through functional analysis. Indeed, this miRNA is involved in the up-regulation of phagocytic activity and in the reduction of inflammatory cytokine production. These data suggested to the authors that this miRNA is involved in early disease pathogenesis. The up-regulation of hsa-miR-146a in PBMCs was confirmed also by Zilahi E et al. and Shi H et al. [16,17]. The first authors investigated both the expression of hsa-miR-146a and its targets IRAK1, IRAK4 and TRAF6 in the PBMCs of 21 SS patients and 10 healthy controls (HCs) using qPCR. Due to the overexpression of TRAF6 in the patients, they concluded that it could be a specific biomarker for this disease. Shi et al. also showed that the overexpression of hsa-miR-146a positively correlated with the VAS (visual analog scale) score for dry mouth, dry eyes and salivary gland swelling. 

A study conducted by Peng L et al. on the Chinese population using microarray analysis has shown the overexpression of hsa-miR-181a in patients compared to HCs [18]. They inferred that it is probably involved in B cells maturation. Interestingly, they found many virus-derived miRNAs dysregulated and this emphasizes the involvement of viral infection in the pathogenesis of SS. To study more specifically the expression profile of miRNAs in B cells, Wang-Renault SF et al. isolated CD19+ B cells from PBMCs using magnetic microbeads [19]. They found five miRNAs differentially expressed in B cells and, in particular, hsa-miR-30b expression seems to be inversely correlated with the expression of B-cell activating factor BAFF. In fact, the inhibition of hsa-miR-30b by transfection experiments resulted in an increased expression of BAFF. BAFF overexpression indeed, was observed in SS, and is associated with B-cell tolerance disruption and increased autoantibody production [20]. 

An important role in the immune responses is played by conventional dendritic cells (cDC) that interact with CD4+ T cells, known to be central in the pathogenesis of autoimmune diseases such as SS [21]. For this reason, Lopes AP et al. decided to evaluate the miRNA’s expression in the subset cDC2s isolated from PBMCs [22]. Using microarray analysis, they found that the expression of hsa-miR-130a and hsa-miR-708 decreased in these cells from patients. Then they investigated the targets of these miRNAs using a proteomics approach based on the use of transfected cells. Regarding hsa-miR-130a, they identified a new gene target, called MSK1, which is a mediator of proinflammatory cytokines, whose expression increased in SS patients. The aberrant expression of the pro-inflammatory IL-17 is critical for the pathogenesis of autoimmune diseases, including SS [23]. Wang J et al. demonstrated that IL-17 is regulated by hsa-let-7d-3p [24]. They quantified the expression of hsa-let-7d-3p using qPCR in CD4+ T cells of SS patients and HCs. They demonstrated that patients showed an aberrant expression of this miRNA and also that it was negatively correlated with IL-17 expression. In addition, they overexpressed or knocked down hsa-let-7d-3p expression in isolated CD4+ T cells in order to validate the correlation between these molecules.

### 3.2. miRNAs Dysregulated in Salivary Glands

Studies regarding miRNAs expression in SS used different types of samples such as salivary gland tissue that has a central involvement in this disease.

Among the complications of SS disease, the worst is the development of non-Hodgkin’s lymphoma [25]. Being able to identify SS patients with higher risk to develop this type of tumor could be very important. Kopsogeorgou EK et al. analyzed the expression of hsa-miR-200b-5p that they supposed was an early predictive marker of susceptibility to develop lymphoma [26]. Using qPCR, they investigated the expression in the minor salivary gland of three groups of patients (without lymphoma, pre-lymphoma and SS-associated lymphoma). They showed that the hsa-miR-200b-5p levels are reduced in patients who have or will develop lymphoma. In addition, another interesting result was that this miRNA was able to discriminate patients with and without lymphoma. 

Although the immune pathways are the most studied in SS, it is known that the calcium signaling pathway modulates Type I Interferon (IFN) signaling and is involved in the pathogenesis of this disease. Jang SI et al. highlighted the link between these two pathways using a smRNA-seq to study the expression of different miRNAs in the epithelial cells of the salivary glands [27]. Among the many altered miRNAs, they focused their attention on hsa-miR-1248. In fact, by functional analysis, they showed that this miRNA had a dual functional role: activating IFN-B production and regulating calcium throughout the down-regulation of its target. 

Yan T et al. analyzed differentially expressed miRNAs in different histological stages of labial minor salivary gland tissue [28]. These authors pointed out two particular miRNAs: hsa-miR-18a whose levels progressively increases along the advanced histological stages and hsa-miR-92a whose levels, on the contrary, decreases. The interesting observation is that these miRNAs are part of the same cluster (hsa-miR-17-92) and it would be interesting to test their combined use as a diagnostic marker. 

Another studied miRNA is hsa-miR-142, known to be involved in T cell functionality. Cortes-Troncoso J et al. investigated the role of this miRNA in glandular lesions and its ability to be transported by exosomes in the glands [29]. First, they saw that the expression of hsa-miR-142 is up-regulated in salivary gland lesions and then they predicted the target molecules (SERCA2B, RyR2, AC9) that this miRNA regulates. Since, among these targets, there are proteins implicated in Ca^2+^ signaling and cAMP production, they hypothesized an involvement of hsa-miR-142 in these pathways. Functional analysis showed that Ca^2+^ signaling and cAMP production are disrupted in hsa-miR-142-3p–transfected human submandibular gland cells and human-derived primary salivary gland epithelial cells. The distinctive feature of SS is the presence of infiltration of leukocytes in the exocrine glands which leads to the apoptosis of tissue cells [30]. 

Among the miRNAs involved in apoptosis there are hsa-miR-1207-5p and hsa-miR-4695-3p which targeted the pro-apoptotic gene TRIM21. Studies performed by Yang et al. showed that these miRNAs are down-regulated and TRIM21 is up-regulated in the salivary glands of SS patients [31]. To evaluate the effects of these miRNAs, human salivary gland cells were transfected with miRNA-mimics and it has been demonstrated the repression of pro-apoptotic gene expression. A recent study conducted by Sembler-Møller ML et al. explored the expression of different miRNAs in 24 patients and 16 HCs using qPCR considering different samples (saliva, salivary gland tissues and plasma) [32]. They identified 15 differentially expressed miRNAs and, among these, the most significantly altered were: hsa-miR-17 family that is decreased in saliva and hsa-mir-29a that is increased in salivary glands.

### 3.3. miRNAs Dysregulated in Other Tissues

In light of the fact that most of the work was done considering the salivary glands, Kim YJ et al. decided to conduct a study on a representative sample of the lacrimal glands, which is the other main tissue involved in the SS [33]. For this purpose, they collected samples of tears. Then they analyzed the expression of 43 miRNAs on tear samples of 18 SS patients and 8 HCs using qPCR. They found many miRNAs differentially expressed between patients and controls. In particular, miRNAs found up-regulated in patients were hsa-miR-16-5p, hsa-miR-34a-5p, hsa-miR-142-3p and hsa-miR-223-3p, while those down-regulated were hsa-miR-30b-5p, hsa-miR-30c-5p, hsa-miR-30d-5p, hsa-miR-92a-3p, hsa-miR-134-5p, hsa-miR-137, hsa-miR-302d-5p, hsa-miR-365b-3p, hsa-miR-374c-5p, and hsa-miR-487b-3p. 

Another study conducted by Pilson Q et al. took into account the primary human conjunctival epithelial cells: on these cells, they performed a microarray analysis in order to evaluate the differences in miRNAs expression between cases and controls [34]. By this analysis, it emerged an interesting result that is the increase of hsa-miR-744-5p expression. This miRNA has as target PELI3, a gene that negatively regulates inflammation. The authors performed functional studies and, as they expected, they confirmed that when hsa-miR-744 was up-regulated, the expression level of PELI3 decreased. Interestingly, a decrease of chemokines CCL5 and CXCL10 was also observed, using an antagomir (a small synthetic RNA that is perfectly complementary to the specific miRNA target, used to inhibit the activity of specific miRNAs). This study highlighted a new role for hsa-miR-744.

## 4. Roles of lncRNAs in SS

In SS, studies in PBMCs and salivary gland tissue identified differentially expressed lncRNAs. Microarray analysis and quantitative real-time PCR (qPCR) are the most used approaches to identify SS-related lncRNAs. Using these approaches, altered expression of several lncRNAs has been detected in various cell types of SS patients. These lncRNAs regulate specific pathways, leading to the inflammation typical of SS.

### 4.1. lncRNAs Dysregulated in PBMCs

One of the first studies focusing on this issue considered the expression of TMEVPG1; it locates on the opposite DNA strand to Interferon γ (IFN-γ) coding gene and it is expressed in CD4+ and CD8+ T cells [35]. Using qPCR, Wang J et al. detected the relative expression of TMEVPG1 in CD4+ isolated T cells of 25 SS patients and 25 healthy controls (HC), showing its up-regulation in patients [36]. In addition, by examining the correlation between the expression of TMEVPG1 and some autoantibodies of SS patients, they showedz that the level of TMEVPG1 was correlated with antibodies anti-SSA, ESR (erythrocyte sedimentation rate) and IgG. Moreover, they demonstrated that the expression level of TMEVPG1 was linked to the proportion of Th1 cells that are involved in the pathogenesis of SS.

Inamo J et al. studied the expression of lncRNAs, using microarray analysis, specifically in peripheral B cells isolated from PBMC samples of 6 SS patients and 6 HCs and validated the results with qPCR in a validation cohort of 12 SS patients and 12 HCs. They identified a lncRNA, LINC00487 that is up-regulated in B-cell subsets of SS patients compared with those of HCs [37]. A key role in the pathogenesis of SS is the IFN signaling [38] and they found that the upstream regulator of LINC00487 in B cells is exactly IFNα. In addition, the expression level of LINC00487 is correlated with the disease score. 

Another lncRNA that is correlated with the disease score and that is central to the regulation of immune response, is NEAT1. Its expression has been evaluated by Ye L et al. in a cohort of 20 SS patients and 10 HCs, using qPCR [39]. They found that the expression of NEAT1 is increased in peripheral T cells compared to HCs. They also explored the role of lncRNA NEAT1 in the pathogenesis of SS carrying out loss-and-gain-of-function experiments. Using this approach, they found that NEAT1 is a positive regulator of SS, in fact NEAT1 knockdown results in the suppression of the expression of inflammatory factors such as TNFα and CXCL8. 

In the SS immunopathogenesis, the population of lymphocytes more strongly involved is that of CD4+ T lymphocytes, as in other autoimmune diseases. CD4+ T cells are highly present in the salivary glands and are responsible for the typical lesions of SS patients [40]. These cells are also overactivated in the peripheral blood. In another expression study, Fu J et al. chose to investigate the expression profile of lncRNA PTV1, which was the most up-regulated lncRNA in the salivary glands [41]. For this purpose, they isolated CD4+ T cells from PBMC samples of SS patients and observed that PTV1 was significantly up-regulated also in these cells. This lncRNA is involved in the proliferation of T cells and in the glycolytic metabolism, essential for the activation of CD4+ T cells. In particular, PTV1 could sustain the expression of Myc, implicated in the reprogramming of glycolysis. They observed that the inhibition of glycolysis could attenuate the disease progression of SS. Therefore, they hypothesized that PTV1 could be connected with the pathogenesis of SS. 

Peng Y et al. used RNA-seq technology to better understand the role of lncRNAs in the PBMC of SS patients [42]. They analyzed 1772 lncRNAs and, among those that were differently expressed, they chose to validate 11 lncRNAs through qPCR. They demonstrated that NRIR, BISPR, LINC00426 and CYTOR were up-regulated in SS patients, while TPTEP1 was down-regulated. In addition, they carried out co-localization and co-expression analysis in order to detect if these lncRNAs, differently expressed, were correlated with mRNAs involved in the immune response. The results showed that these lncRNAs are involved in numerous pathways that contribute to the pathogenesis of the disease, such as NF-κB signaling pathways, MAPK signaling pathways, JAK-STAT signaling pathway.

Finally, due to the ability of lncRNAs to bind miRNAs, it is interesting to study the interaction between these two groups of molecules and how, together, they modulate genes. Based on this, Dolcino M et al. first analyzed the expression profile of more than 50,000 lncRNAs, by microarray analysis, in a cohort of 8 SS patients and 8 HCs; then they carried out a network analysis resulting in the identification of lncRNA-miRNA-gene interactions [43]. Therefore, they obtained six deregulated lncRNAs. and, among these, they selected LINC00657, LINC00511 and CTD-2020K17.1 because of their targeting activity against the highest number of genes involved in SS pathogenesis. In particular, these 3 lncRNAs regulate some miRNAs that played a role in B-cell development [43]. It is known that B cells are involved in the pathogenesis of SS. In particular, CD27+ memory B cells, marginal zone B cells, plasmablasts and plasma cells were observed as altered in the peripheral blood and salivary glands of patients with pSS [38].

### 4.2. lncRNAs Dysregulated in Salivary Gland

Given the central involvement of salivary glands in SS, the examination of the expression profile of lncRNAs in this tissue is interesting. Using microarray analysis, for the first time Shi H et al. identified 1243 differentially expressed lncRNAs in labial salivary glands of four SS patients [44]. Then, they validated the expression of eight lncRNAs in a cohort of 30 SS patients and 16 HCs, using qPCR. They confirmed that LINC00426-003, AC017002.1, n336161, NR_002712, LINC02384, lnc-UTS2D-1:1, n340599 and TCONS_l2_00014794 are up-regulated in SS patients. In addition, they observed a strong correlation between these lncRNAs and clinical characteristics, such as disease course and immunological disorders.

### 4.3. Genetics of lncRNAs

There are very few studies concerning the variability of lncRNAs genes in SS, compared to other autoimmune diseases. In an association study conducted on the Chinese Han population, the H19 rs2839698 and rs3741219 polymorphisms were examined by Huang A et al. [45]. No significant evidence was detected for the relationship of H19 polymorphisms and risk of this disease. In another association study conducted on Italian population, Colafrancesco S et al. showed an interesting association between a polymorphism in HCP5 lncRNA and SS susceptibility [46]. They analyzed rs3099844 (C > A) polymorphism in 195 SS patients and 248 HCs and they showed for the first time that the frequency of rs3099844 CA and AA genotypes in SS patients was significantly higher than in the HC. Interestingly, HCP5 was not only associated with susceptibility to SS, but also with a higher risk to develop a specific clinical phenotype. In particular, it was associated with immunological disorders, hypergammaglobulinemia, leukopenia and lymphoma (that is the worst severe clinical manifestation of SS).

## 5. Conclusions

It is now widely demonstrated that epigenetics could play an important role in the pathogenesis of autoimmune diseases, such as SS. Among the epigenetic mechanisms that regulate gene expression, non-coding RNAs (miRNAs and lncRNAs) are involved in the modulation of different pathways and biological processes. Moreover, lncRNAs and miRNAs interact with each other forming a complex network of gene expression regulation. 

SS is a complex disease whose underlying pathogenic processes are still partly unknown. As shown in this review, different studies highlighted that both miRNAs and lncRNAs present a dysregulation in the expression level in SS patients; these observations could help to clarify the etiopathogenesis of this disorder since they are involved in crucial pathways. In Table 1, we reported all the studies that have investigated the miRNAs and lncRNAs expression in SS.

Concerning miRNAs, numerous studies have discovered unique sets of miRNAs that may play a critical role in SS disease pathogenesis; while the expression studies of lncRNAs in SS are only starting to emerge. The advent of high throughput technologies and the availability of public databases have facilitated the recognition of the complicated interplay among lncRNAs, miRNAs and target genes. The major limitation of the mentioned studies is their small sample size and the lack of validation of their results in independent cohorts of patients. 

Further research is required to better understand the molecular mechanisms involved in the regulation of these non-coding RNAs. At the same time, it will also be important to analyze the patient’s sub-phenotypes in order to better understand and clarify disease mechanisms underlying the diverse clinical manifestations. These studies could have consequences also in diagnostic and therapeutic fields. An example of translation in the clinical practice is the use of miRNAs as biomarkers able to recognize early patients at higher risk to develop more severe forms; as shown in Table 1, hsa-miR-200b has been identified by the authors as a novel promising marker predicting lymphoma development in patients with SS [26]. Since the development of non-Hodgkin’s lymphoma is the worst complication of SS disease, it could be useful to analyze the levels of hsa-miR-200b in large-scale samples in order to verify its alteration and to suggest its possible use as a clinical biomarker. 

Regarding lncRNAs, although the results here mentioned are very promising, many of them have been reported only in a single study, therefore it is still premature, at present, to consider lncRNAs as biomarkers in SS. 

Moreover, since gene variants may exert regulatory effects on gene expression, it could be interesting to increase studies to identify SNPs (single nucleotide polymorphisms) in miRNA and lncRNA genes, possibly associated with SS and its clinical manifestations. This kind of studies could also give a contribution to the development of a more personalized medicine approach in SS treatment.

## Figures and Tables

**Table 1:** List of principal miRNAs and lncRNAs potentially involved with SS pathogenesis.

**Tissues**	**miRNAs**	**Evidence**	**Refs.**
PBMCs	hsa-miR-146a	up-regulated	[15,16,17]
	hsa-miR-181a	up-regulated	[18]
	hsa-miR-30b	down-regulated	[19]
	hsa-miR-130a	down-regulated	[22]
	hsa-miR-708	down-regulated	[22]
	hsa-let-7d	up-regulated	[24]
Salivary glands	hsa-miR-200b	down-regulated in patients who have or will develop lymphoma	[26]
	hsa-miR-1248	up-regulated	[27]
	hsa-miR-18a	up-regulated	[28]
	hsa-miR-92a	down-regulated	[28]
	hsa-miR-142	up-regulated	[29]
	hsa-miR-1207	down-regulated	[31]
	hsa-miR-4695	down-regulated	[31]
	hsa-miR-29a	up-regulated	[32]
Saliva	hsa-miR-17 family	down-regulated	[32]
Lacrimal glands	hsa-miR-16	up-regulated	[33]
	hsa-miR-34a	up-regulated	[33]
	hsa-miR-142	up-regulated	[33]
	hsa-miR-223	up-regulated	[33]
	hsa-miR-30b,c,d	down-regulated	[33]
	hsa-miR-92a	down-regulated	[33]
	hsa-miR-134	down-regulated	[33]
	hsa-miR-137	down-regulated	[33]
	hsa-miR-302d	down-regulated	[33]
	hsa-miR-365b	down-regulated	[33]
	hsa-miR-374c	down-regulated	[33]
	hsa-miR-487b	down-regulated	[33]
Conjunctiva	hsa-miR-744	up-regulated	[34]
**Tissues**	**lncRNAs**	**Evidence**	**Refs.**
PBMCs	TMEVPG1	up-regulated	[36]
	LINC00487	up-regulated	[37]
	NEAT1	up-regulated	[39]
	PTV1	up-regulated	[41]
	NRIR	up-regulated	[42]
	BISPR	up-regulated	[42]
	LINC0042	up-regulated	[42]
	CYTOR	up-regulated	[42]
	TPTEP1	down-regulated	[42]
	LINC00657	up-regulated	[43]
	LINC00511	down-regulated	[43]
	CTD-2020K17.1	up-regulated	[43]
Salivary glands	LINC00426-003	up-regulated	[44]
	AC017002.1	up-regulated	[44]
	n336161	up-regulated	[44]
	NR_002712	up-regulated	[44]
	LINC02384	up-regulated	[44]
	lnc-UTS2D-1:1	up-regulated	[44]
	n340599	up-regulated	[44]
	TCONS_l2_00014794	up-regulated	[44]

## Data Availability

Not applicable.
